# The Contribution of Artificial Intelligence in Nursing Education: A Scoping Review of the Literature

**DOI:** 10.3390/nursrep15080283

**Published:** 2025-08-01

**Authors:** Federico Cucci, Dario Marasciulo, Mattia Romani, Giovanni Soldano, Donato Cascio, Giorgio De Nunzio, Cosimo Caldararo, Ivan Rubbi, Elsa Vitale, Roberto Lupo, Luana Conte

**Affiliations:** 1 Città di Lecce Hospital, Gruppo Villa Maria, 73100 Lecce, Italy; fcucci@gvmnet.it (F.C.); giansoldano@gmail.com (G.S.); 2 Health Governance Agency of the Calabria Region-Azienda Zero, 88100 Catanzaro, Italy; dario.marasciulo@regione.calabria.it; 3 IRCCS, Hospital San Raffaele, 20132 Milano, Italy; romani.mattia@hsr.it; 4 Department of Physics and Chemistry, University of Palermo, 90128 Palermo, Italy; donato.cascio@unipa.it; 5 Laboratory of Biomedical Physics and Environment, Department of Mathematics and Physics, “E. De Giorgi”, University of Salento, 73100 Lecce, Italy; giorgio.denunzio@unisalento.it; 6 Laboratory of Advanced Data Analysis for Medicine (ADAM) at DReAM, University of Salento and ASL (Local Health Authority), “V. Fazzi” Hospital, 73100 Lecce, Italy; 7 National Institute for Nuclear Physics (INFN), 73100 Lecce, Italy; 8 School of Nursing, University of Salento, 73100 Lecce, Italy; cosimo.caldararo@unisalento.it; 9 School of Nursing, University of Bologna, 48018 Faenza, Italy; ivan.rubbi@auslromagna.it; 10 Directorate of Health and Nursing Professions, ASL (Local Health Authority), 70123 Bari, Italy; vitaleelsa00@gmail.com; 11 “San Giuseppe da Copertino” Hospital, Local Health Authority, 73043 Copertino, Italy; roberto.lupo@asl.lecce.it

**Keywords:** artificial intelligence, nurse, education, nursing

## Abstract

**Background and Aim:** Artificial intelligence (AI) is among the most promising innovations for transforming nursing education, making it more interactive, personalized, and competency-based. However, its integration also raises significant ethical and practical concerns. This scoping review aims to analyze and summarize key studies on the application of AI in university-level nursing education, focusing on its benefits, challenges, and future prospects. **Methods:** A scoping review was conducted using the Population, Concept, and Context (PCC) framework, targeting nursing students and educators in academic settings. A comprehensive search was carried out across the PubMed, Scopus, and Web of Science databases. Only peer-reviewed original studies published in English were included. Two researchers independently screened the studies, resolving any disagreements through team discussion. Data were synthesized narratively. **Results:** Of the 569 articles initially identified, 11 original studies met the inclusion criteria. The findings indicate that AI-based tools—such as virtual simulators and ChatGPT—can enhance students’ learning experiences, communication skills, and clinical preparedness. Nonetheless, several challenges were identified, including increased simulation-related anxiety, potential misuse, and ethical concerns related to data quality, privacy, and academic integrity. **Conclusions:** AI offers significant opportunities to enhance nursing education; however, its implementation must be approached with critical awareness and responsibility. It is essential that students develop both digital competencies and ethical sensitivity to fully leverage AI’s potential while ensuring high-quality education and responsible nursing practice.

## 1. Introduction

Artificial intelligence (AI) is defined as the ability of machines to perform tasks that would typically require human intelligence, including reasoning, learning, planning, and creativity. In recent years, technological advancements, increased computing power, and the availability of vast datasets have driven the rapid expansion of AI applications across various sectors [1,2,3,4], including nursing education [5]. In this context, AI is not just a digital aid but a transformative element capable of reshaping how nursing students gain knowledge, develop clinical skills, and handle complex care scenarios. Generative AI tools, such as chatbots and large language models like ChatGPT, are emerging as innovative educational resources. These technologies help learners by providing theoretical insights, practical guidance, and interactive experiences, such as explanations, simulations, and decision-making exercises [6]. As highlighted by Narayanasamy et al., AI-powered chatbots are increasingly being explored for their potential to support nursing students’ learning, communication, and clinical decision-making. However, concerns remain regarding pedagogical alignment, ethical use, and faculty preparedness [7].

In particular, the use of virtual patients and AI-based simulators has shown benefits in enhancing communication skills and the ability to manage realistic clinical scenarios. These tools provide students with safe, interactive, and immersive learning environments that replicate real-world conditions [8]. A systematic review of AI-based teaching strategies in nursing found that approaches such as adaptive quizzes, AI-assisted case generation, and automated feedback loops are increasingly being utilized to support formative assessment and foster critical thinking [9].

Moreover, AI enables the customization of educational pathways by adapting content and pacing to individual learning needs. Personalized feedback systems can monitor student progress and deliver real-time interventions to enhance learning outcomes. Liu et al. reported that AI-enhanced personalized learning improves academic performance, engagement, and student satisfaction [10]. These benefits are particularly relevant in nursing, where learners vary widely in experience and learning styles.

However, the integration of AI into nursing education is not without challenges. Ethical and practical issues remain prominent. Concerns include the quality and representativeness of data used to train AI algorithms [11], the protection of student privacy, and the potential for AI to inadvertently reinforce inequalities or biases [12]. In addition, both faculty and students may exhibit resistance to the adoption of unfamiliar technologies, which can hinder effective implementation [13].

Furthermore, global health strategies have underscored the urgency of embedding digital competencies—AI literacy included—within health education curricula. International organizations, such as the World Health Organization and the International Council of Nurses, recognize that preparing future healthcare professionals for digital transformation requires educational systems to evolve accordingly [14,15].

The need for a conscious and responsible use of AI places additional responsibility on educators. They must not only help students grasp AI’s practical applications but also encourage critical awareness of its ethical dimensions [11,16]. Moreover, gaps in digital literacy among faculty and the fast pace of AI development create barriers to the effective use of these technologies in both education and clinical practice [17]. In this context, it is essential to investigate the current state of AI integration in nursing education. A scoping review was considered the most suitable approach due to the emerging nature of this topic and the heterogeneity in study designs and outcomes. This methodology enables the systematic mapping of existing evidence, identification of key themes, and recognition of gaps in the literature. The aim of this scoping review is to explore and summarize empirical studies focused on the use of AI in nursing education, with particular attention to its application in university programs and its potential contribution to training competent, patient-centered nurses.

## 2. Methods

### 2.1. Protocol and Registration

This scoping review was registered on the Open Science Framework (OSF) platform on 2 February 2025, https://doi.org/10.17605/OSF.IO/VUE4R.

### 2.2. Eligibility Criteria

This scoping review was conducted following the Population, Concept, and Context (PCC) framework to guide the selection of relevant studies (Table 1). The target population comprised research involving nursing students and nurse educators. Several types of studies were excluded from this review, including editorials, commentaries, expert opinions, non-peer-reviewed sources, discussions on contemporary issues, qualitative interviews, conference proceedings, meeting abstracts, systematic reviews, narrative reviews, integrative reviews, scoping reviews, retracted articles, and conference papers. Only studies published in English were included. This review specifically explored the integration of AI in nursing education, emphasizing studies conducted within an academic environment. The academic context was defined as formal educational environments where nursing education is delivered, including university undergraduate and graduate programs (e.g., bachelor’s, master’s, and doctoral levels), as well as recognized professional training institutions.

In this review, “artificial intelligence” (AI) was operationally defined as any computer-based system capable of mimicking human cognitive functions, such as learning, problem-solving, reasoning, or decision-making. This includes machine learning algorithms, natural language processing systems (e.g., ChatGPT), virtual patient simulations, and adaptive tutoring systems. Traditional digital tools such as standard learning management systems (LMSs), static e-learning platforms, or non-adaptive chatbots were excluded unless they incorporated autonomous learning or decision-making components typical of AI.

### 2.3. Informational Sources

A comprehensive search was conducted across multiple bibliographic databases, including PubMed, Scopus, and Web of Science (WOS), to identify relevant studies for this review. These databases were selected because they represent the most comprehensive and multidisciplinary sources for health sciences and educational research. PubMed covers the biomedical literature extensively, while Scopus and Web of Science offer broad coverage of interdisciplinary and nursing education journals. This combination ensured a balanced and exhaustive search across clinical, technological, and pedagogical domains. Two researchers (F.C. and L.C.) independently performed the queries. In cases of uncertainty or disagreement regarding study inclusion, issues were discussed within the research team until a consensus was reached. To refine the dataset, duplicate records were identified and removed. Furthermore, the reference lists of key studies and relevant reviews were manually examined to capture any significant studies that may have been missed.

### 2.4. Search Strategy

The search strategy employed a blend of specific keywords and phrases, combined with Boolean operators (AND, OR), to ensure a comprehensive and systematic collection of relevant studies.

Both free-text terms and structured subject headings (e.g., title/abstract terms) were incorporated where applicable. Keywords associated with AI (e.g., “artificial intelligence,” “machine learning,” “deep learning”) were combined with terms related to nursing, education, and implementation to encompass all the relevant literature (Table 2).

### 2.5. Data Items

Data on article characteristics (e.g., authorship, publication year, country of origin), population details (e.g., participant count, course year), findings, and conclusions were systematically gathered.

Data extraction was performed independently by two reviewers (F.C. and L.C.) using a standardized data charting form specifically developed for this review. Any discrepancies between reviewers were discussed and resolved through consensus, with the involvement of a third reviewer (R.L.) when necessary. The extracted data were used to construct a descriptive summary of the included studies, focusing on the benefits, challenges, and future perspectives of applying AI in nursing education. A narrative synthesis approach was employed to present and organize the results.

Stakeholder consultation was not conducted as part of this review, in accordance with the optional nature of this step in the JBI methodology.

In accordance with JBI guidance for scoping reviews, no critical appraisal of individual sources of evidence was performed, as the aim was to map the existing literature rather than assess study quality.

## 3. Results

A total of 569 records were identified through database searches, including 511 from PubMed, 3 from Scopus, and 55 from WOS. No records were identified from other sources or registries. Prior to screening, 16 duplicate records were removed, resulting in 553 unique records for screening.

**Figure 1:**
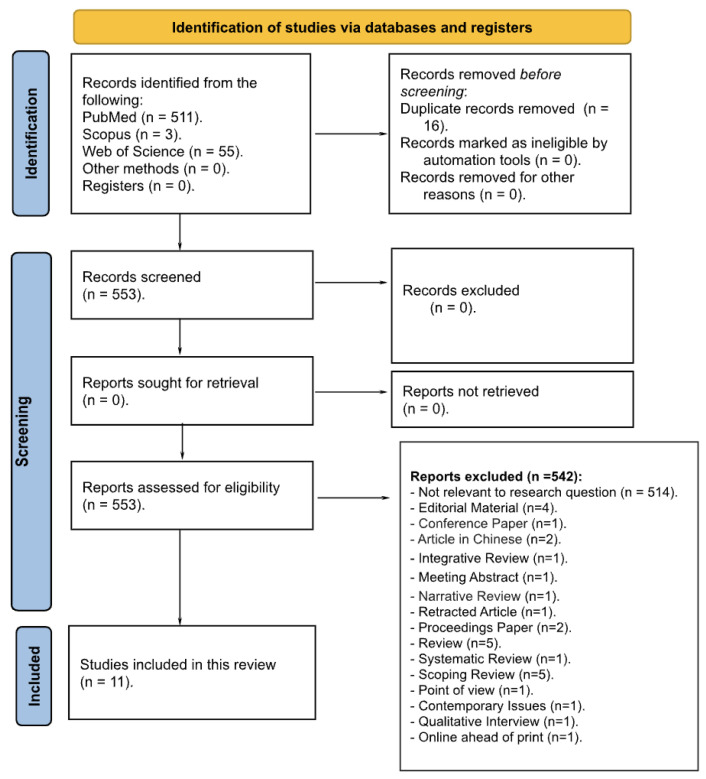
PRISMA 2020 flow diagram [18] of the research.

The titles and abstracts of these 553 records were reviewed, leading to the exclusion of 514 records that were not relevant to the research question. Specifically, 232 articles were excluded because they did not address artificial intelligence as defined in the eligibility criteria, while 282 were excluded because, despite referring to AI, they did not meet one or more elements of the PCC framework (Population, Concept, Context), such as not focusing on nursing education, not being original research, or falling outside the academic context. Additionally, 28 full-text articles were excluded for various reasons, as outlined in the PRISMA 2020 flow diagram (Figure 1). Ultimately, 11 studies met the inclusion criteria and were included in the final analysis.

### 3.1. Characteristics of Sources of Evidence

This scoping review includes 11 original research articles, each analyzing the role and influence of AI in nursing students’ education. Specifically, the benefits, challenges, and future perspectives of this technology, as well as its impact on skill development during university training for future nurses, were examined. The articles included in this review were published between October 2019 and January 2025.

### 3.2. Results of Individual Sources of Evidence

The included studies are collected and summarized in Table 3. The table provides a comprehensive overview of the study development settings involving various international universities. Additionally, it shows the elements analyzed for conducting each individual study.

## 4. Discussion

### 4.1. Definitional Challenges in AI Educational Research

In this review, we adopted a broad and inclusive definition of artificial intelligence (AI), operationally described as any computer-based system capable of mimicking human cognitive functions such as learning, problem-solving, or reasoning. Although this approach aligns with perspectives in several prior reviews, such as Buchanan et al. [30], it raises important conceptual challenges. For example, tools like virtual patient (VP) simulations often rely on pre-programmed decision trees rather than adaptive or learning algorithms and, therefore, may not technically qualify as AI. This definitional ambiguity reflects a broader issue in the field: the lack of a shared, technically grounded understanding of what constitutes AI in educational contexts. As evidenced by Zawacki-Richter et al. [31], diverse digital tools are frequently labeled under the umbrella of “AI” despite operating on fundamentally different principles. Consequently, our review captures a wide spectrum of technological complexity—from simple rule-based systems to advanced generative models such as ChatGPT. This heterogeneity underscores the need for future research to clearly specify the functional capabilities of the technologies under investigation and to adopt more precise classifications. Clarifying these distinctions will strengthen the rigor and interpretability of future evidence in nursing education.

To address this heterogeneity, we propose a simple conceptual framework that classifies the AI technologies identified in this review into three main categories:

**Generative**—AI Systems: These include tools based on large language models (e.g., ChatGPT), used primarily for content generation, writing assistance, or dialogic interaction.
**Adaptive Learning Platforms**—These systems personalize educational content based on learner performance, often using machine learning techniques. They are typically integrated into learning management systems or digital teaching environments.
**Simulation-Based Systems**—These include virtual patient simulations or interactive clinical scenarios that may be rule-based or enhanced with AI functionalities and are designed to support clinical reasoning and communication training.

This classification helps clarify the composite nature of the technologies included and allows for a more structured interpretation of their educational impacts.

### 4.2. Educational Benefits and Student Perceptions

The use of AI in nursing education is gaining increasing attention due to its potential to enhance the effectiveness and efficiency of learning. AI-driven technologies can transform nursing training [29], making it more interactive, personalized, and data-driven. It has been demonstrated that nursing students using AI systems, such as ChatGPT, report improvements in learning outcomes, clinical practice, and overall satisfaction [23]. However, these results are primarily based on self-reported measures—such as student reflections and satisfaction surveys—and not always corroborated by objective performance indicators. For instance, Gonzalez-Garcia et al. [23] reported statistically significant improvements in students’ perceived preparedness and engagement, although no clear gain in academic performance was observed. Similarly, Arcia [22] found that students appreciated the integration of generative AI in coursework while also emphasizing the need for clearer assignment instructions and more robust assessment tools. These AI-based tools foster critical thinking and enhance student engagement through innovative learning modalities [32]. Bumbach [28] emphasized how ChatGPT facilitated brainstorming and clinical reasoning exercises, particularly in graduate-level assignments, although the outcomes were not formally evaluated. AI can also enhance communication skills and decision-making abilities through virtual patients (VPs) and simulation environments. Shorey et al. [24] found that such tools increased students’ confidence in clinical interactions with patients, families, and multidisciplinary teams, although the outcomes were assessed qualitatively through student feedback. In contrast, Simsek-Cetinkaya and Cakir [21] used a randomized controlled design to compare AI-assisted and traditional simulations in breast self-examination training, showing greater satisfaction but reduced technical performance in the AI group. A positive attitude toward AI integration has been reported in multiple studies. Labrague et al. [26] emphasized the importance of cultivating openness to AI during university training to improve patient care outcomes. Similarly, Lukić et al. [20] found that students view AI positively in the context of professional preparation, particularly when technologies are clearly aligned with learning objectives and accompanied by adequate training. However, both studies relied on attitude scales rather than learning outcomes, highlighting a gap between perception and measurable impact. AI contributes to personalized learning experiences and fosters readiness for complex healthcare environments and interprofessional collaboration [28]. Recent findings confirm that nursing students exposed to AI-assisted teaching show improved engagement and readiness for clinical tasks, especially when AI is embedded in scenario-based learning or combined with reflective debriefing [33]. Moreover, Abujaber et al. demonstrated that incorporating AI technologies into nurse education increases student confidence, autonomy, and critical thinking, particularly in developing countries where such tools can help overcome faculty shortages [34].

### 4.3. The Research–Practice Gap in AI Integration

Our systematic search yielded only 11 empirical studies that met the inclusion criteria. This limited number of original research articles stands in stark contrast to the large volume of opinion pieces, commentaries, and conceptual discussions found during the screening process. Such a discrepancy suggests that the integration of AI in nursing education remains under-researched at an empirical level. This research–practice gap is particularly relevant given the growing presence of AI tools in healthcare and higher education. The scarcity of rigorous studies documenting actual implementation, outcomes, and challenges may hinder informed adoption by educators and institutions. This underscores the need for more primary research focused on real-world educational practices, user experiences, and long-term impacts of AI tools. We believe this review contributes to addressing this gap by mapping current empirical efforts and highlighting areas where future investigation is both needed and feasible.

### 4.4. Risks, Limitations, and Ethical Considerations

While AI tools offer significant opportunities to enhance nursing education, they are accompanied by important limitations that must be acknowledged and addressed [25]. One of the main risks concerns the emotional and psychological impact on students: Simsek-Cetinkaya and Cakir [21] found that AI-driven simulations increased student satisfaction but also heightened anxiety during virtual activities, a result confirmed by other studies [35,36]. In parallel, ethical concerns remain central, especially in relation to data privacy, the preservation of academic integrity, and the potential devaluation of the nursing degree, as highlighted by Summers et al. [19]. These risks underline the urgent need for institutional and national policies that define ethical guidelines for the use of AI, ensuring that its integration supports—not compromises—educational quality [37]. Furthermore, several authors stress the importance of equipping faculty with the skills needed to critically evaluate, implement, and supervise AI tools in academic settings [38]. Without adequate educator training, the integration of AI risks becoming superficial or misaligned with pedagogical goals. Krueger et al. [27] emphasized the importance of preventing misuse and ensuring the responsible adoption of AI, while El Arab et al. [25] noted that institutions must engage both students and faculty in shared strategies for its effective application. To ensure the safe and effective use of AI, students must not only be trained in its applications but also understand its risks [22]. Open dialogue between educators and learners is essential to encourage a balanced and reflective approach to AI use [39].

### 4.5. Policy Implications and Future Directions

Awareness of both the benefits and limitations of AI is essential to ensure its ethical implementation and preserve the integrity of the educational process. From a policy perspective, integrating AI into nursing curricula requires institutional and national guidelines addressing ethical standards, data privacy, and faculty readiness. Such policies should support educator training and promote equitable access to AI tools across institutions [13,30,40,41,42]. Preparing nursing students today means equipping future professionals with the competencies necessary to safely integrate AI into clinical practice.

### 4.6. Limitations

This scoping review presents several limitations that should be considered when interpreting the results. First, the selection of databases may have led to the exclusion of relevant studies not indexed in PubMed, Scopus, or Web of Science. In addition, the inclusion of only English-language publications may have resulted in the omission of valuable research published in other languages, thereby limiting the global applicability of the findings. The included studies also varied widely in terms of AI technologies, research designs, and educational settings. This heterogeneity made it difficult to conduct a uniform synthesis and reduced the comparability of findings across contexts. Moreover, all the studies analyzed were conducted in high-income countries, such as the United States, Australia, Singapore, Croatia, and Spain. This geographic concentration limits the generalizability of results to low- and middle-income settings, where access to AI technologies, digital infrastructure, and faculty training may differ significantly. Furthermore, recent scoping reviews of generative AI in education have also noted a complete absence of studies from low- and lower-middle-income countries, highlighting marked gaps in our understanding of socio-economic and cultural influences on AI adoption [43,44]. Finally, the rapid pace of technological advancement in AI presents a temporal limitation. Some of the included studies reference technologies or tools that may already be considered outdated in 2025. This temporal gap may affect the relevance and applicability of earlier findings. Ongoing research and regular updates will be essential to align the evidence base with current and emerging developments in AI.

## 5. Conclusions

The integration of artificial intelligence (AI) in nursing education offers transformative opportunities to make learning more interactive, personalized, and data-driven, thereby enhancing both theoretical knowledge and clinical competencies among students. Technologies such as virtual patients (VPs) and generative systems like ChatGPT have shown potential to improve student engagement, boost confidence, and support the development of communication and decision-making skills during clinical training. However, these benefits must be balanced with critical attention to ethical, technical, and pedagogical risks. Concerns persist regarding data privacy, academic integrity, and the appropriate use of AI tools in educational settings. Therefore, it is crucial that nursing curricula integrate not only technological tools but also structured frameworks for their responsible use. Faculty development plays a pivotal role in this process: educators must be equipped with the knowledge and skills necessary to effectively evaluate, implement, and supervise AI-based learning resources. Equally important is ensuring equitable access to AI technologies across diverse institutions and student populations. From a policy perspective, the development of institutional guidelines and national strategies is essential to ensure that the integration of AI supports innovation without compromising educational quality. In summary, AI represents a promising resource for both the present and the future of nursing education. To fully realize its potential, it is essential to address the ethical, pedagogical, and infrastructural challenges and to support educators and students through informed, inclusive, and forward-thinking approaches.

## Figures and Tables

**Table 1:** The Population, Concept, and Context (PCC) tool for conducting this scoping review.

Population	Nursing students enrolled in undergraduate, graduate, or licensed practical nurse (LPN) programs, as well as nurse educators involved in formal academic instruction.
Concept	Use of artificial intelligence (AI) technologies in nursing education, including machine learning algorithms, natural language processing systems (e.g., ChatGPT), adaptive tutoring systems, and virtual patient simulators.
Context	Formal academic environments such as universities, professional nursing schools, and structured continuing education programs within accredited institutions.

**Table 2:** Combination of keywords used with the Boolean operator.

PubMed	( artificial intelligence [Title/Abstract] OR machine learning [Title/Abstract] OR deep learning [Title/Abstract] OR AI [Title/Abstract] OR A.I. [Title/Abstract]) AND (use [Title/Abstract] OR usage [Title/Abstract] OR utilis [Title/Abstract]) AND (learn [Title/Abstract] OR teach [Title/Abstract] OR study [Title/Abstract] OR develop [Title/Abstract] OR education [Title/Abstract] OR training [Title/Abstract]) AND (nursing [Title/Abstract] OR nurse [Title/Abstract] OR student nurses [Title/Abstract] OR pre-registered nurses [Title/Abstract] OR nursing students [Title/Abstract]) )
Scopus	( TITLE-ABS-KEY (artificial intelligence OR machine learning OR deep learning OR AI OR A.I.) AND TITLE-ABS-KEY (use OR usage OR utilis) AND TITLE-ABS-KEY (learn OR teach OR study OR develop OR education OR training) AND TITLE-ABS-KEY (nursing OR nurse OR student nurses OR pre-registered nurses OR nursing students) )
Web of Science	( TI= (artificial intelligence OR machine learning OR deep learning OR AI OR A.I.) AND TI= (use OR usage OR utilis) AND TI= (learn OR teach OR study OR develop OR education OR training) AND TI= (nursing OR nurse OR student nurses OR pre-registered nurses OR nursing students) )

**Table 3:** Summary of articles.

References	Title	Article Type	Sample Size	AI Technology Used	Type of AI Technology	Learning Dimension Supported	Educational Outcomes	Summary	Country
[19]	Navigating challenges and opportunities: Nursing student’s views on generative AI in higher education.	Qualitative study	13 nursing students	Generative AI tools (e.g., ChatGPT 4.0)	Generative AI	Ethical awareness, critical thinking, AI literacy	Nursing students recognize both the benefits and challenges of generative AI, highlighting educational impact, ethics, equitable access, and the need for safe, practical integration in nursing education.	A study conducted at a University in Australia. Thirteen interviews with nursing students were conducted, focusing on six themes: Educational Impact of AI Tools; Equitable Learning Environment; Ethical Considerations; Technology Integration; Safety and Practical Utility; and Generational Differences.	Australia
[20]	First-year nursing students’ attitudes towards artificial intelligence: Cross-sectional multi-center study.	Cross-sectional multicenter study	336 first-year nursing students	AI-powered systems for nursing care	Adaptive Learning	Attitudes toward AI, perceived usefulness	Nursing students are generally positive about AI but need targeted education to overcome practical reservations.	A study conducted at four Croatian universities that involved 336 first-year nursing students. The General Attitudes towards AI Scale, consisting of 20 Likert-type items, was used.	Croatia
[21]	Evaluation of the effectiveness of artificial intelligence assisted interactive screen-based simulation in breast self-examination: An innovative approach in nursing students.	Randomized controlled trial	103 first-year nursing students	AI-assisted, screen-based simulations	Simulation-Based	Practical skills (breast self-examination), satisfaction, anxiety management	AI-assisted simulation increases student satisfaction but also raises anxiety and is less effective than standard simulation for breast self-examination skills.	A study conducted at a Turkish university. A total of 103 nursing students were enrolled to assess the effectiveness of an AI-assisted, interactive, screen-based simulation for breast self-examination.	Turkey
[22]	Incorporation of Generative AI in an Introductory Nursing Informatics Course.	Case study	37 nursing students	Generative AI tools (e.g., ChatGPT)	Generative AI	Understanding of AI use, ethics, risk awareness	Integrating generative AI in a nursing course improved student understanding of its uses, risks, and ethics, although assignment instructions need refinement.	A study conducted at a U.S. university. AI training was introduced in an introductory nursing informatics course, and the written reflections of 37 students were subsequently analyzed.	USA
[23]	Impact of ChatGPT usage on nursing students education: A cross-sectional study.	Research article	98 nursing students	Generative AI tools (e.g., ChatGPT)	Generative AI	Learning performance, satisfaction, digital readiness	ChatGPT improves nursing student learning, satisfaction, and performance, supporting tech adoption and better preparation for future healthcare settings.	A study conducted at a university in Spain. A total of 98 students were enrolled. Using three validated questionnaires, the study evaluated the impact of ChatGPT on nursing students’ training and learning outcomes.	Spain
[24]	A Virtual Counseling Application Using Artificial Intelligence for Communication Skills Training in Nursing Education: Development Study.	Development study	150 Year-2 and Year-3 nursing undergraduates	NLP chatbots with 3D virtual avatars	Simulation-Based	Communication skills, confidence	Virtual patients can boost nursing students’ communication confidence but require further development to effectively simulate real-life interactions.	A study conducted at a university in Singapore. A three-dimensional (3D) virtual patient (VP) avatar was developed and tested to better prepare students for interactions with patients, families, and other healthcare professionals.	Singapore
[25]	Tool or Tyrant: Guiding and Guarding Generative Artificial Intelligence Use in Nursing Education.	Research article	95 university educators	Generative AI tools (e.g., ChatGPT)	Generative AI	Critical thinking, ethical evaluation, policy awareness	Efficiency gains, critical thinking benefits, ethical/transparency concerns, need for AI literacy and guidelines.	A total of 95 university educators in the U.S. were enrolled in the study. Through a SWOT analysis, the educators provided insights on the strengths, opportunities, weaknesses, and threats of using AI in nursing education.	USA
[26]	Factors influencing student nurses’ readiness to adopt artificial intelligence (AI) in their studies and their perceived barriers to accessing AI technology: A cross-sectional study.	Cross-sectional study	323 nursing students	AI-powered technologies	Adaptive Learning	Readiness for AI adoption, perceived barriers	Student nurses show moderate readiness to adopt AI but need improved tech skills, AI knowledge, and practical experience to overcome access barriers.	A total of 323 students from the Philippines were enrolled in the study. Three items from the AI scale developed by [27] were used to assess the nursing students’ readiness to adopt AI technology, explore associated factors, and identify perceived barriers to accessing AI.	Philippines
[27]	ChatGPT in Higher Education: Practical Ideas for Addressing Artificial Intelligence in Nursing Education.	Abstract	N/A	AI detection software/tools	Generative AI	Academic integrity, AI policy awareness	Establishing integrity-focused policies and using AI-detection tools fosters ethical AI use and reinforces academic honesty in nursing education and practice.	The article explored the role of academic institutions and programs in promoting AI adoption in nursing education.	USA
[28]	The Use of AI Powered ChatGPT for Nursing Education.	Abstract	N/A	Generative AI tools (e.g., ChatGPT)	Generative AI	Instructional design, technology-enhanced learning	Nursing educators gain knowledge about ChatGPT and can design assignments leveraging it to enhance student learning experiences despite limitations.	The article examined how academic institutions and programs promote AI adoption in healthcare and nursing education.	USA
[29]	The use of artificial intelligence for graduate nursing education: An educational evaluation.	Abstract	N/A	Generative AI tools (e.g., ChatGPT)	Generative AI	Practical application, innovation in patient care tools	An AI-based assignment helped graduate nursing students become familiar with ChatGPT, applying it to develop practical patient care tools; it was well received and clarified the best practices for AI use in nursing.	In the study conducted in Florida, students received training in AI/chatbot technology and were subsequently assigned a task to complete using ChatGPT.	USA

AI: artificial intelligence; SWOT: Strengths, Weaknesses, Opportunities, and Threats. N/A = Not Available.

## Data Availability

The original contributions presented in this study are included in this article. Further inquiries can be directed to the corresponding author.

## Public Involvement Statement

There was no public involvement in any aspect of this research.

## Guidelines and Standards Statement

This manuscript was drafted following the PRISMA-ScR (Preferred Reporting Items for Systematic reviews and Meta-Analyses extension for Scoping Reviews) guidelines for scoping review research.
